# Artificial Adaptive Systems and predictive medicine: a revolutionary paradigm shift

**DOI:** 10.1186/1742-4933-7-S1-S3

**Published:** 2010-12-16

**Authors:** Enzo Grossi

**Affiliations:** 1 Centro Diagnostico Italiano, Milano, Italy

## Abstract

An individual patient is not the average representative of the population. Rather he or she is a person with unique characteristics. An intervention may be effective for a population but not necessarily for the individual patient. The recommendation of a guideline may not be right for a particular patient because it is not what he or she wants, and implementing the recommendation will not necessarily mean a favourable outcome.

The author will describe a reconfiguration of medical thought which originates from non linear dynamics and chaos theory. The coupling of computer science and these new theoretical bases coming from complex systems mathematics allows the creation of “intelligent” agents able to adapt themselves dynamically to problem of high complexity: the Artificial Adaptive Systems, which include Artificial Neural Networks( ANNs ) and Evolutionary Algorithms ( EA).

ANNs and EA are able to reproduce the dynamical interaction of multiple factors simultaneously, allowing the study of complexity; they can also help medical doctors in making decisions under extreme uncertainty and to draw conclusions on individual basis and not as average trends. These tools can allow a more efficient Technology Transfer from the Science of Medicine to the Real World overcoming many obstacles responsible for the present translational failure. They also contribute to a new holistic vision of the human subject contrasting the statistical reductionism which tends to squeeze or even delete the single subject sacrificing him to his group of belongingness. A remarkable contribution to this individual approach comes from Fuzzy Logic, according to which there are no sharp limits between opposite things, like health and disease. This approach allows to partially escape from probability theory trap in situations where is fundamental to express a judgment based on a single case and favours a novel humanism directed to the management of the patient as individual subject.

## Background

Physicians every day try to accomplish their mission: to give the better care and assistance to their individual patients. They know perfectly that an individual subject is typically “unique”. He or she has specific characteristics, qualities and features, different perceptions of risk value, different values scales, different familiar environments and social roles, all together interacting in a complex way. In other words no subject is equivalent to another, nor even mono-ovular twins.

But physician has to take decisions continuously, in front to every single individual, facing always with uncertainty. Will my diagnosis be confirmed later on? Will my treatment work in this patient? Will this patient follow my indications? These are questions that most physicians ask themselves every day.

The fundamental resource for them still remain their experience and their intelligence in using experience as a guide.

Unfortunately for them current medical practice has created an explosion of information, which per se represent a burden for a busy medical doctor.

Classical statistics predictive models like discriminant analysis, logistic regression, etc., are able to utilize a number of factors simultaneously higher than a human mind. This number generally ranges between 8-15 variables. In reality medical doctors ca easily collect much more information. It is not unusual to have at hand, especially when faced with treatment planning for a chronic degenerative disorder, hundreds of different variables, consisting of clinical history data, objective findings, symptomatology, multi-item scales of different meanings, laboratory examinations, imaging procedures etc. With the increased availability and use of functional genomics and digital imaging we now tentatively have at our disposition thousands of data per subject.

This new de facto reality has created yet another paradox: in comparison with 10-20 years ago we are now able to collect more data per subject than subjects per study.

More features imply more information and potentially higher accuracy. Unfortunately an important paradox is that more features we have, the more difficult information extraction is. In this high dimensional space, the hyper points corresponding to single individuals are sparse and the notion of proximity fails to retain its meaningfulness. For this reason clustering become extremely hard to be performed. In this situation we are dealing with flat, rectangular data set, a sort of telescope data set. This kind of data set are intractable from a traditional statistics point of view due to the fact that the excessive amount of degrees of freedom allows any kind of data interpolation most of the time meaningless.

A part from quantitative features, Non linearity, complexity, fuzzy interaction are new emerging qualitative features of chronic degenerative diseases which account for most morbidity and mortality in western world. Unfortunately even the most powerful and well established statistical methods were developed in the first half of the past century when the scenario was dominated by acute infective diseases and the available information was much more simple, or at maximum “complicated” rather than “complex” in comparison with today.

There is now the reason to ask a fundamental question: is the mathematics used in medicine what it should be given the complexity of the chronic degenerative diseases?

Complexity is based on small elementary units working together in small populations of synchronous processes. In a complex system each component changes, over time, losing its identity outside of the system. Complexity needs a different kind of mathematics, able to handle chaotic behaviour, non linear dynamics, and fractal geometry [1,2]. There are a number of different reasons to apply complex systems mathematics on predictive medicine and some of them are listed in the table 1.

**Table 1:** Motivations to apply complex systems mathematics on predictive medicine

Processes are based on complex networks of interacting genes and proteins.
Health status is the consequence of dynamic processes that regulate these networks
Non linear critical thresholds link to pathology
The predictions have to be applied at individual patient level.
Huge amount of data per subject hamper statistical tests

The use of computers has opened the floodgates to methods of data collection that were impossible just a decade ago, solving the quantitative problem of information load, but computers are also responsible for permitting computationally intensive medical analyses with newer numerical algorithms addressing the qualitative challenge; this is sometimes called computational and mathematical medicine.

Newer statistical approaches, base on new mathematical and logic assumptions broadly belonging to artificial adaptive system family and complex theory setting allow to tame these intractable data sets. Seen in this perspective computer science is now playing the role which mathematics did from the seventeenth through the twentieth centuries: providing an orderly, formal framework and exploratory apparatus for knowledge progress.

Actually the coupling of computer science and these new theoretical bases coming from complex systems mathematics allows the creation of “intelligent” agents able to adapt themselves dynamically to problem of high complexity: the Artificial Adaptive Systems, which include Artificial Neural Networks( ANNs ) and Evolutionary Algorithms ( EA).

## Artificial Adaptive Systems

ANNs and EA are able to reproduce the dynamical interaction of multiple factors simultaneously, allowing the study of complexity.

ANN and EA are adaptive models analyzing data which are inspired by the functioning processes of the human brain and of evolution respectively . They are systems which are able to modify their internal structure in relation to a function objective. They are particularly suited for solving problems of the non linear type, being able to reconstruct the approximate rules that put a certain set of data - which describes the problem being considered - with a set of data which provides the solution (ANN) or to reconstruct the optimal data for a given set of rules or constraints (EA). For a detailed description of these models and tools we refer to recent reviews [3,4]

### Artificial Neural Networks

Artificial Neural Networks are adaptive models for the analysis of data which are inspired by the functioning processes of the human brain. They are systems which are able to modify their internal structure in relation to a function objective. They are particularly suited for solving problems of the non linear type, being able to reconstruct the approximate rules that put a certain set of data - which describes the problem being considered - with a set of data which provides the solution.

**Figure 1:**
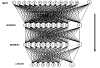
Example of Supervised ANN.

The base elements of the ANN are the nodes, also called processing elements (PE), and the connections (Figure 1). Each node has its own input, from which it receives communications from other nodes and/or from the environment and its own output, from which it communicates with other nodes or with the environment. Finally, each node has a function f through which it transforms its own global input into output.

Each connection is characterized by the strength with which pairs of nodes are excited or inhibited. Positive values indicate excitatory connections, the negative ones inhibitory connections.

The connections between the nodes can modify themselves over time. This dynamic starts a learning process in the entire ANN. The way through which the nodes modify themselves is called “Law of Learning”. The total dynamic of an ANN is tied to time. In fact, for the ANN to modify its own connections, the environment has to necessarily act on the ANN more times. Data are the environment which acts on the ANN.

The learning process is, therefore, one of the key mechanisms that characterize the ANN, which are considered adaptive processing systems. The learning process is one way to adapt the connections of an ANN to the data structure that make up the environment and, therefore, a way to “understand” the environment and the relations that characterize it.

### Evolutionary algorithms

At variance with neural networks which are adaptive systems able to discover the optimal hidden rules explaining a certain data set, Evolutionary Algorithms(EA) are Artificial Adaptive Systems able to find optimal data when fixed rules or constraints have to be respected. They are in other words optimisation tools which become fundamental when the space of possible states in a dynamic system tends toward infinitum.

An EA for example can help to distribute the original sample in two or more sub-samples with the aim of obtaining the maximum performance possible from an ANN that is trained on the first sample and tested on the second. It is possible, in order to limit eventual optimistic polarizations in the evaluation of the performance, to invert the two samples and to consider the mean between the two approximations obtained as fitness of the algorithm and as an estimate of the model’s quality.

Also the problem to select among the different variables available those most related to a particular outcome without the recur to linear correlation parameters can be approached with EA. When linear systems are used, an instrument exists, correlation index , which indicates the degree of relationship existing between the input and output variables of the system, in this way suggesting which of the variables available to use to build a model of the problem. The problem of selecting a subset of variables on which to build the model for the process under examination stems from the fact that, when data are gathered to build the Data Base, the relationship between the collected variables and the function of the process being examined is not known. In this case the natural approach is to include all of the variables that may have a connection with the event being studied. The result of this approach is that often a series of variables which do not contain any information regarding the process being examined are present. These variables, inserted in the model, cause an increase of the noise and therefore a greater difficulty for the ANN to learn the data correctly.

The coupling of ANN and EA and brings to the concept of artificial organisms, able to optimize the classification performance and prediction ability.

Training & Testing (T&T) and Input Selection (I.S.) which have been combine in a single system called TWIST [5,6] are example of such artificial organisms which are devoted to the problems above discussed respectively.

## Basic philosophy of Artificial Adaptive Systems

The basic principle which is proposed in AAS is very simple: all the biological signals from all the sources available are analyzed together -and not individually- both in time and space. The reason for such an approach is quite simple and self-explaining: the instant value of the system in any recording source depends, in fact, upon its previous and following values (how many, and in which amount for each previous state?), upon the previous and following values of all the other recording sources (how many, and in which amount for each previous state?).

In summary, the aim of the “analyzer” is not to analyze the language of each individual variable, but to evaluate the meta-language which considers the holistic contribution of all the recorded variables. We, in fact, believe that the equilibrium of each individual subject is defined by a specific background signal model, distributed in time and in the space. Such a model is a set of background invariant features able to specify the quality of the immune activity for example. The system that we propose to apply in this research context completely ignores the subject’s contingent characteristics. It utilizes a recurrent procedure which squeezes at progressive steps the significant signal and progressively eliminates the non-significant noise.

## The paradigm shifts of Artificial Adaptive Systems in predictive medicine

The use of Artificial Adaptive Systems and in particular Neural Networks are already emerging as new trends in medical statistics. Although these methods are not yet in widespread use, they have already had a clinical impact in specific areas, notably cervical cytology, x-Ray mammography and early detection of acute myocardial infarction where large-scale prospective multicenter studies have been carried out. An extensive review on this subject has been published by Lisboa [7].

There are in the literature many examples of successful application of ANN in outcome research.

Our group has proved the usefulness of the added predictive value gained with the use of advanced artificial neural networks coupled with evolutionary algorithms in a number of medical fields, ranging from heart diseases, gastroenterology and neurology with special regard to Alzheimer disease, stroke and Amyotrophic Lateral Sclerosis ([8-15].

AAS bring a number of revolutionary paradigm shifts which will have a strong impact in predictive medicine. They are listed in the table 2.

**Table 2:** Paradigm shift introduced by AAS in medicine

No limitation in the amount of data processed
No limitation in the different nature of data processed
No limitation in the degree of complexity of data processed
Bottom - up computation: models are data driven
Interactions among different factors are easily picked-up
Inference takes place at individual level
Internal validity of modelling ensured with validation protocols
Fuzzy logic allows to escape from the probability theory trap

AAS are able to reproduce the dynamical interaction of multiple factors simultaneously, allowing the study of complexity; this is very important for the researcher interested to deep the knowledge of a specific disease or to better understand the possible implications relative to strange associations among variables. This has to do to what is called “intelligent data mining”. But one the other hand AAS can also help medical doctors in making decisions under extreme uncertainty and to draw conclusions on individual basis and not as average trends. The modern patient wants to be treated as an individual person and not just as a statistics. Patients want to know their own risk, not just a parameter regarding a class of people similar to them just for some aspects. AAS are very powerful in modelling at single individual level, and by combining several parallel AAS trained on the same data set is possible to make multiple statistics on a single subject, allowing in this way the calculation of the confidence interval of the prediction estimate. Finally AAS make possible to treat huge amount of information without squeezing arbitrarily the data and without loosing complexity. This contributes to a new holistic vision of the human subject contrasting the statistical reductionism, which tends to squeeze or even delete the single subject sacrificing him to his group of belongingness. A remarkable contribution to this individual approach comes from Fuzzy Logic, according to which there are no sharp limits between opposite things, like health and disease. This approach allows to partially escape from probability theory trap in situations where is fundamental to express a judgment based on a single case and favours a novel humanism directed to the management of the patient as individual subject.

## List of abbreviations

AAS: Artificial Adaptive Systems; ANN: Artificial Neural Networks; EA: Evolutionary Algorithms

## Competing interests

The author declares no competing interests.
